# Mindfulness-Based Program Embedded Within the Existing Curriculum Improves Executive Functioning and Behavior in Young Children: A Waitlist Controlled Trial

**DOI:** 10.3389/fpsyg.2019.02052

**Published:** 2019-09-10

**Authors:** Philip Janz, Sharon Dawe, Melissa Wyllie

**Affiliations:** School of Applied Psychology, Griffith University, Mount Gravatt, QLD, Australia

**Keywords:** executive function, mindfulness, children, teachers, education, curriculum

## Abstract

There is a growing evidence base for mindfulness-based interventions in educational settings. Notably, there has been little investigation of the potential benefits of classroom-based mindfulness programs in children in the early school years (Preparatory/Kindergarten, Grades 1 and 2) despite early childhood being a period characterized by the development of self-regulation and executive functions. The present study investigated the effects of a mindfulness program that was embedded within a school curriculum. This waitlist controlled trial investigated the effects of a mindfulness program, CalmSpace, delivered by classroom teachers across two school terms. A total of 55 students, *M*_*age*_ = 76.4 months, *SD* = 8.62, were allocated to participate in CalmSpace in Terms 3 and 4. Thirty-six students in the waitlist control condition, *M*_*age*_ = 80.53 months, *SD* = 13.04, participated in the intervention in Term 4. The start of Term 3 served as baseline (Time 1), and measures were obtained at the end of Term 3 (Time 2) and the end of Term 4 (Time 3). Direct measures of executive functioning using the Flanker Inhibitory Control and Attention Test (Flanker Task) and Dimensional Change Card Sort Task (DCCS) from the National Institute of Health Toolkit were obtained. Teachers’ report of children’s behavior was also obtained using the Strengths and Difficulties Questionnaire (Teacher version) at the beginning and at the end of Term 3, and at the end of Term 4. Children who received the CalmSpace program showed improvements on the DCCS relative to waitlist control at Time 2 (Cohen’s *d* = 0.48) and Time 3 (Cohen’s *d* = 1.10). Similar results were found on the Flanker Task with greater improvements found at Time 2 (Cohen’s *d* = 0.77) and Time 3 (Cohen’s *d* = 1.33). Teachers reported improvements for those receiving CalmSpace at Time 2 on total SDQ scores, Emotional Symptoms, Conduct Problems, Hyperactivity/Attention (Cohen’s *d* = 0.32, 0.14, 0.46, 0.30, 0.33, and 0.53, respectively) compared to waitlist control and at Time 3 (Cohen’s *d* = 0.85, 0.37, 0.48, and 0.90, respectively). The findings demonstrate that implementing the CalmSpace program can lead to improvements in EF and attention for young children. Despite limitations, this study provides promising evidence that the inclusion of focused, targeted mindfulness activities throughout the day may represent a value-added component to the regular school curriculum that can result in benefits for the students.

## Introduction

Executive functions (EFs) consist of three, interrelated core skills: inhibitory control, working memory, and cognitive flexibility (Miyake et al., 2000; Diamond, 2013). From these, higher-order EFs such as reasoning, problem-solving, and planning are built (Lunt et al., 2011; Collins and Koechlin, 2012). These highly complex processes develop from birth through to young adulthood, before declining in older age (Casey et al., 2000; Steinberg, 2005; Anderson et al., 2011). They help us to think before we act, resist temptations or habitual reactions, stay focused, reason, problem solve, flexibly adjust to changing demands or priorities, and see things from new and different perspectives (Diamond, 2013).

The development of EFs is a key predictor of adaptive functioning, particularly in early and middle childhood (Anderson et al., 2011). The voluntarily inhibition of behavior underlies children’s ability to behave in a socially appropriate manner, and to pay attention to and remember information, while the capacity to flexibly shift between tasks and information is imperative for effective functioning across all domains (Cipriano and Stifter, 2010). These skills have been associated with lower levels of problem behaviors, in both younger and older children, and have been found to correlate with, and predict over time, low levels of negative emotion, better empathy and conscience development, committed compliance, and higher levels of social competence (Raffaelli et al., 2005; Spinrad et al., 2007). Notably, preschoolers with better EF skills learn more from a given amount of instruction and practice (Welsh et al., 2010; Benson et al., 2013; Hassinger-Das et al., 2014; Bascandziev et al., 2016). These foundational skills are strongly associated with children’s social and emotional wellbeing typically assessed using measures of emotional regulation and effortful control (Maynard et al., 2017).

There has been extensive investigation of the developmental trajectory of EF across the lifespan, with converging views that EF skills begin to emerge in the first year of life, and continue to develop throughout childhood, adolescence, and in some areas, into adulthood. Using the three-factor structure of EF proposed by Miyake et al. (2000), Best and Miller (2010) provided an in-depth review of the developmental trajectory of EFs across childhood and adolescence. They highlight the difficulties inherent in any study of EFs, namely the issue of task impurity in which complex tasks involve multiple processes making identification of foundational or pure EF tasks difficult. Thus, understanding the developmental trajectory of EF requires a nuanced and careful analysis of the task requirements in addition to clarity on the theoretical framework that underpins the analysis of developmental trajectory.

With this caveat in mind, there are some key principles that emerge from reviews of the literature and comprehensive factor analytic studies. Assessing the developmental trajectory of inhibitory control, independent of working memory is challenging in young children. For example, widely used inhibition tasks such as the Day/Night task require verbal inhibition of a prepotent response (say Day instead of Night) while the Dimensional Change Card Sort (DCCS) requires the learning of a rule that is subsequently inhibited. Both these, and many other inhibition tasks used in early childhood, have a working memory component (Best and Miller, 2010). Critically, for the current study, improvements in the inhibition of prepotent responding appears in children aged 5–8 years although it is possible that the age for greatest improvement is actually younger (age 3–5 years; Best and Miller, 2010; Anderson et al., 2011).

Unlike inhibition, it appears that working memory continues to improve in a relatively steady manner from preschool to early adolescence. Notably, many working memory tasks have a component of executive control, particularly tasks designed for older children (Best and Miller, 2010). Cognitive flexibility appears to be the last EF to emerge, scaffolded by the earlier development of inhibitory control and working memory (Best and Miller, 2010). A rapid improvement in cognitive flexibility occurs at 5–6 years of age, with development of these abilities continuing to develop across adolescence (Luciana and Nelson, 1998; Huizinga et al., 2006), with the ability to shift accurately and swiftly continuing to improve across adolescence (De Luca et al., 2003). In Montroy et al. (2016) study, the researchers found that there are individual differences in the developmental trajectory of EFs in early childhood, with children falling into three groups: early, intermediate, and late developers. The late developers constituted at least 20% of the sample (there was variability across sites). While EFs are proposed to provide a foundational platform for the development of behavioral self-regulation in these early years, they continue to develop across the lifespan with developmental spurts in mid to late adolescence, with staggered development. Initial increases in reward sensitivity are followed by gains in cognitive control (Gullo and Dawe, 2008) and further development into adulthood as metacognitive skills develop (Best and Miller, 2010).

There is a growing body of research indicating that EFs and relatedly, behavioral self-regulation, can be fostered through interventions that provide children with opportunities to practice their developing EFs at increasing levels (e.g., Rueda et al., 2005; Karbach and Kray, 2009; Thorell et al., 2009; Mackey et al., 2010; Tominey and McClelland, 2011; Neville et al., 2013; Weiland and Yoshikawa, 2013; Schmitt et al., 2015). These interventions often require children to pause momentarily and reflect before responding. In other words, be intentional about their cognition and behavior [see Diamond and Lee (2011) for a review].

Initial evidence suggests that mindfulness training can nurture EFs in children (Harnett and Dawe, 2012; Meiklejohn et al., 2012; Tang et al., 2012; Schonert-Reichl et al., 2015). Mindfulness involves focusing on thoughts, feelings, or perceptions that arise moment-to-moment in a cognitively non-elaborative and emotionally non-reactive, way (i.e., “paying attention in a particular way: on purpose, in the present moment, and non-judgmentally,” Kabat-Zinn, 1994, p. 4). Mindfulness practices intended to cultivate this state of mind typically include meditation exercises, and the bringing of mindful awareness to daily activities such as eating. Being mindful requires the cognitive control strategies described earlier, as EFs can be contrasted with non-conscious attention and acting on “automatic pilot” (e.g., Diamond, 2013). Thus, mindfulness training has been proposed to enhance EFs by targeting top-down executive processes, such as cognitive flexibility and sustained attention, while minimizing the effects of bottom-up influences such as anxiety and stress (Zelazo and Lyons, 2012). This involves training to develop a specific brain state that is consistent with brain activity associated with a quiet alert state, that in turn allows for focused attention (Posner et al., 2013). To date, there has been no direct investigation of the effects of mindfulness training in children or young adults on brain structure and function. In the absence of such data, there has been a case made for extrapolation from adult studies [see Lyons and DeLange (2016) for a review], in which training in mindfulness practices are associated with improvements in brain regions associated with focused attention (Tang et al., 2010, 2012) and emotional regulation (e.g., Farb et al., 2010). Significant gains in inhibitory control are also seen in young children, which arguably are reflected in the capacity to control behaviors such as hitting, slapping, and shouting. Again, in the absence of systematic evaluation in young children, extrapolation from adult studies of impulse control suggests that disinhibition tasks activate dorsolateral prefrontal brain regions (e.g., Vara et al., 2014) and that individuals with disorders characterized by disinhibition such as borderline personality disorder show improvement in these brain regions following mindfulness training.

There is now an established literature reporting on the effectiveness of mindfulness programs delivered in educational settings. Much of this literature has been synthesized within either narrative (e.g., Greenberg and Harris, 2012) or meta-analytic reviews (e.g., Zenner et al., 2014; Zoogman et al., 2014; Maynard et al., 2017). Zoogman et al. (2014) restricted their meta-analysis to studies of youth and is thus of less relevance for the current study. Notably, however, they found that the intervention effect sizes were greater in studies using clinical samples. Zenner et al. (2014) conducted a meta-analysis of 24 school-based mindfulness studies that had been published prior to August 2012. Three of these studies investigated the effectiveness of a mindfulness intervention for children in early primary school (mean age across these studies was 8 years), finding improvements in parent and teacher reports of executive functioning (Flook et al., 2010), direct measures of selective attention (Napoli et al., 2005), and cognitive computer assessment of EFs (Biegel and Brown, 2010). The authors concluded that overall there was good evidence that mindfulness-based interventions were associated with improvements in cognition. A further meta-analysis conducted by Maynard et al. (2017) added to these findings. Sixty-one studies were identified with a publication cut off date of May 2015. Of these, 35 randomized or quasi-experimental studies were included in the meta-analysis. There are three key findings of relevance to the current study. Firstly, significant but small effects were found for mindfulness-based interventions provided to children in school settings on cognitive outcomes (EF, memory, cognition, attention) and socioemotional outcomes (anxiety, stress, emotional regulation, social skills, self-esteem, internalizing behaviors). No effects were found on academic performance (standardized achievement tests, reading, grades, curriculum content knowledge) or on measures of behavior (disciplinary referrals, aggression and other externalizing behaviors, time on task compliance, attendance). Secondly, the meta-analytic findings pointed to a lack of heterogeneity across outcomes (with the exception of behavior) indicating that despite some apparent diversity in the content and delivery of the mindfulness intervention, the outcomes were very similar with respect to cognitive, socioemotional, and academic outcomes. Finally, it was noted that 31% of studies had a teacher-delivered intervention. Although this was only examined as a mediator in one of the analyses, there was no relationship between whether the instructor was a classroom teacher or an independent expert in mindfulness on behavioral outcomes. Notably, only 2 of the 35 studies included in the meta-analysis involved children in the preschool age range. Flook et al. (2010) compared a “Kindness Curriculum” delivered by experienced mindfulness instructors twice a week (20–30 min per lesson). A rigorous multimethod approach to measurement of outcomes was adopted consisting of teacher report, neurodevelopmental assessment of prosocial behavior (sharing), and executive functioning and school grades. Teacher-rated social competence total score was significant as were two subscales: prosocial behavior and emotional regulation. There were no significant differences on measures of executive functioning (drawn from the NIH Toolbox; Gershon et al., 2013) but differences on three of five school grades outcomes. The second study from the Maynard et al. (2017), meta-analysis was conducted by Razza et al. (2015) evaluating a mindful yoga intervention in children (age 3–5 years). One class received the teacher-delivered intervention, the control class did not receive any intervention and could be considered to be a business as usual condition, but notably included a component that aimed to enhance attention and focused listening. There were some differences found on measures of behavioral inhibition (pencil tapping task; attentional impulsivity) and a trend favoring the intervention group on a delay of gratification task and another behavioral inhibition task. There were no differences on parent report of children’s effortful control or two other direct assessments. The small sample size and the potential confounds of delivering the mindful yoga with an adjoining control classroom and a curriculum that already included a focus on supporting the development of focused attention were challenges that resulted in the study lacking adequate statistical power.

Since this comprehensive meta-analysis there have been three further studies evaluating the effectiveness of a mindfulness intervention in young children. Wood et al. (2018) evaluated a mindfulness intervention delivered by specialist facilitators to preschoolers (age 3–5 years; *n* = 12) compared to a control condition (*n* = 15). Outcomes were assessed using a purpose-designed teacher report questionnaire that assessed five components of EF as outlined by Miyake et al. (2000). There were no significant differences on any measure of executive functioning although small sample size and lack of validated measures may have reduced the opportunity to determine if the program was helpful. Zelazo et al. (2018) found that children who were randomly assigned to a mindfulness plus reflection training program significantly outperformed the business-as-usual (BAU) group in direct behavioral assessments of EFs, with differences most pronounced at the 4–6 week follow-up testing. Off (2018), in an unpublished dissertation, undertook an evaluation of MindUP in children (*N* = 159) in 15 Kindergarten classrooms across eight schools using a pre–post design. There were significant improvements in children’s internalizing behaviors and resiliency but no changes in externalizing behaviors. These findings were not moderated by gender or grade (early vs. late Kindergarten).

To date, much of the research investigating the effects of mindfulness training for children has focused on middle childhood and adolescence, with relatively little research examining mindfulness training with children in Preparatory/Kindergarten and Grades 1 and 2. It does not appear that mindfulness-based interventions are more effective when delivered by teachers rather than external experts (Maynard et al., 2017); therefore, we developed a curriculum that could be embedded within the school day and delivered by classroom teachers. Thus, if successful, there was greater opportunity for embedding this approach into other educational settings without reliance on external resources and additional funds. Accordingly, the purpose of the present study was to investigate the effects of a mindfulness-based program delivered through the classroom curriculum to young children using a waitlist-controlled design. Given there has been growing focus on the importance of ensuring mindfulness programs are delivered with fidelity (Domitrovich and Greenberg, 2000; Durlak and DuPre, 2008), a second purpose was to measure implementation fidelity when implemented in a “real-world” setting delivered by school teachers in primary school classrooms.

## Materials and Methods

### Participants

One hundred and fifteen students were given consent forms to take home to parents/carers. Of these 91 children returned consent forms across Preparatory Year, Year 1 and Year 2 classes (*M*_*age*_ = 78.03 months, *SD* = 10.71): CalmSpace mindfulness program, *n* = 55 (34 boys and 21 girls); waitlist group, *n* = 36 (12 boys and 24 girls). The school is located in a regional area in Queensland and serves a local population of 1898 people. The area is identified as being in the first quintile of Socio Economic Area, placing it in the lowest 20% of areas in Australia (Australian Bureau of Statistics), with nearly a third of people born outside of Australia. The school itself has a population of children that broadly represent this demographic with 8% of students identified as Indigenous Australian, and 23% had a language background other than English (Australian Curriculum, Assessment and Reporting Authority [ACARA], 2017). There were significant differences between the two groups on gender (Table 1).

**TABLE 1:** Participant demographics and scores at baseline.

		**Intervention (*n* = 55)**	**Waitlist (*n* = 36)**	**Total (*n* = 91)**
Age, *M* (*SD*)		76.4 (8.62)	80.53 (13.04)	78.03 (10.71)
Female^∗^ (*n*; %)		21; 38%	24; 67%	45; 49%
English as second language (*n*; %)		16; 29%	5; 14%	21; 23%
Flanker scores				
	Range	61–123	73–134	61–134
	Mean (*SD*)	96.25 (11.26)	96.86 (12.42)	96.43 (11.73)
DCCS scores				
	Range	67–129	51–130	51–130
	Mean (*SD*)	95.49 (13.28)	98.61 (16.29)	96.51 (14.23)
SDQ scores				
	Range	0–24	0–21	0–24
	Mean (*SD*)	7.76 (6.59)	5.86 (5.34)	6.99 (6.20)
	% scoring > borderline clinical cut off (=16)	11%	11%	11%

*^∗^Significant gender differences at baseline, *t*(89) = 2.737, *p* = 0.007.*

### Design

**FIGURE 1:**
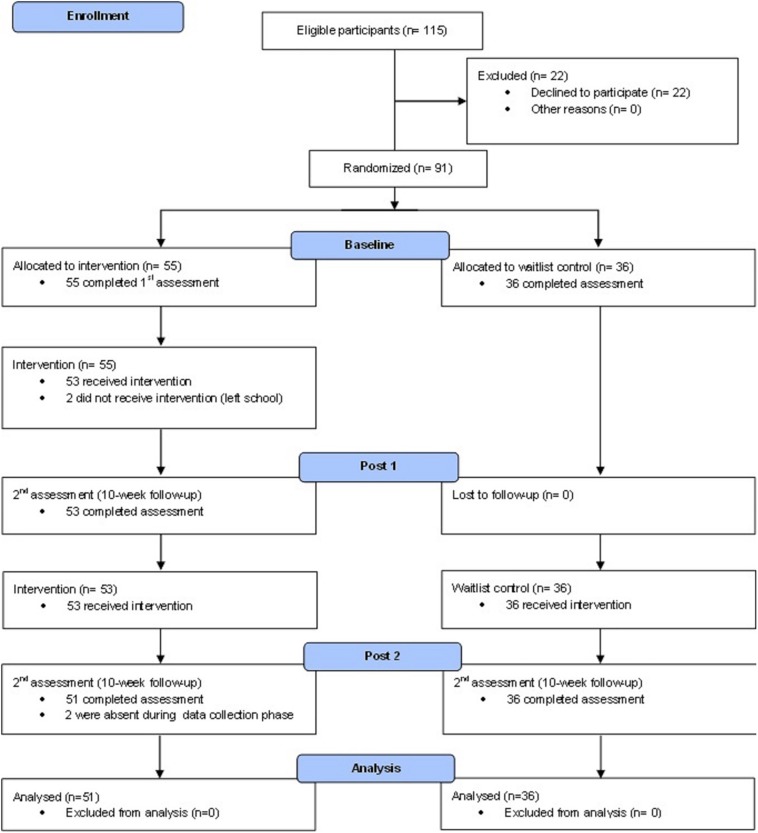
Consort flowchart of recruitment and study condition allocation.

Classes within the participant primary school were randomly allocated via a hat draw to participate in CalmSpace in both Terms 3 and 4, 2017, or placed on a waitlist for Term 3 and commence participation in CalmSpace in Term 4, 2017. Classes randomly allocated to CalmSpace Terms 3 and 4 consisted of a single preparatory class of 18 children; a Grade 1 class of 24 children and a composite Grades 1 and 2 class of 26 children. Classes randomly allocated to the waitlist control group consisted of a single preparatory class of 17 children and a Grade 2 class of 28, Allocation to conditions and study throughput is shown in Figure 1 (one child did not complete measures of EF).

### Measures

#### Executive Function

##### NIH toolbox: flanker inhibitory control and attention test ages 3–7 years (flanker task) (Gershon et al., 2013)

The Flanker Task measures a participant’s ability to pay attention to a specific target while inhibiting attention to irrelevant task dimensions. The test requires the participant to focus on a central directional target which is flanked by stimuli to the left and right (fish in the case of children in this age range). The task is to indicate the direction of the middle fish when there are either congruent flankers (i.e., flanker fish are pointing in the same direction) or incongruent flankers (flanker fish are pointing in the opposite direction). The task takes approximately 3 min to administer.

Scoring is obtained using the age-corrected standard score. For the purposes of this study, the accuracy score and the reaction time score were analyzed separately. Each score obtained can be compared to those in the NIH Toolbox representative age-matched normative sample, mean = 100, *SD* = 15. Higher scores indicate greater accuracy and faster reaction time. Convergent and construct validity with other measures of EF have been established for the flanker task (Weintraub et al., 2013).

##### NIH toolbox: dimensional change card sort test (DCCS) ages 3–7 years (Gershon et al., 2013)

The DCCS is a measure of cognitive flexibility or executive shifting. It measures the capacity to plan, organize, and monitor the execution of behaviors that are strategically directed in a goal-oriented manner (Gershon et al., 2013). Two target pictures are presented that vary along two dimensions (e.g., shape and color). Participants are asked to match a series of bivalent test pictures (e.g., yellow balls and blue trucks) to the target pictures. First, participants are asked to complete five “pre-switch” trials according to one dimension (e.g., color) and then, if the participant succeeds in four of the five test trials, the program advances to the “post-switch,” a set of five trials on the other dimension (e.g., shape). If the participant meets the criteria of getting four or more out of five trials correct on the post-switch they will complete the mixed block (switch trials), which consists of 30 shape/color trials. In the “switch” trials the participant must change the dimension being matched. For example, after four straight trials matching on shape, the participant may be asked to match on color on the next trial and then revert back to shape, thus requiring the cognitive flexibility to quickly choose the correct stimulus.

As with the Flanker Task, scoring is based on a combination of accuracy and reaction time and interpreted using age-matched norms, with higher scores indicating greater accuracy and faster reaction times. The test takes approximately 4 min to administer. Convergent and construct validity with other measures of EF have been established (Weintraub et al., 2013).

#### Teacher Report

##### Strengths and difficulties questionnaire – teacher form (SDQ)

The SDQ (Goodman and Scott, 1999) is a widely used behavioral screening questionnaire that correlates highly with other measures of behavior problems including the Child Behavior Checklist (Goodman and Scott, 1999; Koskelainen et al., 2000). The 25 items are divided between five scales: emotional symptoms (five items), conduct problems (five items), hyperactivity/inattention (five items), peer relationship problems (five items), and prosocial behavior (five items). The SDQ has been used extensively throughout the world (Goodman, 1997; Woerner et al., 2004; Vostanis, 2006) and Australia (Vostanis, 2006). The Teacher Form was used for the present study, which asks teachers to rate the behavior of children in their classroom. All five subscales and a “total difficulties score” were used.

#### Measures of Implementation

To monitor fidelity and dosage, the CalmSpace program teachers were given a daily CalmSpace checklist in which they were to track and record their daily implementation of the CalmSpace program. Two components of intervention implementation were assessed in the present study. The first component consisted of the “core practice” of attending to the sound of a gong at points of major transition, such as beginning the school day or returning from lunch. This was a compulsory activity that the teachers implemented three times a day, every day. The second component was an additional 10 mindfulness-based activities (Table 2) provided to teachers, who were then able to use their own professional judgment as to which activities from this list would be more beneficial for the classroom. The implementation of the additional activities was recorded separately from the core practice. Teachers were provided with a daily implementation checklist and were asked to complete the checklist each day by ticking which activities were delivered that day.

**TABLE 2:** Description of activities in the CalmSpace program.

**Activity**	**Description**	**Target of activity**
The gong	The gong, which is delivered daily three times a day (beginning, middle, and end of the day), gives students a daily practice that they can use that helps them to focus and relax their minds enabling them to pay closer attention to what is happening inside them and around them in their environment.	Focused attention and a state of calmness.
	A gong is sounded in the classroom. This is a melodic sound and each day a different child was permitted to bang the gong. Children were instructed to listen to the gong until the sound disappeared.	
Balloon belly breathing	The teacher asks the students to get into the “Mindful Bodies.” The teacher asks the children to imagine a balloon in their belly that getters bigger and bigger throughout out their big breath in, then deflates slowly as they breathe out. Children were asked to take three big breaths and blow up their belly balloon, focusing on their breathe and calming the mind.	Focused attention, state of calmness, and cognitive flexibility.
Body scan	This 5 min exercise helped children to calm down and relax, with a view to developing increased body and emotion awareness. Children sat in their chairs with their eyes closed while the teacher talked through a script provided by the researchers. The script starts by bringing children’s attention to the top of their head and moves down through the body to the toes, asking children to focus on this body part while they take a slow breath.	Focused attention, state of calmness, and cognitive flexibility.
Change of activity	Children were encouraged to take a “mindful moment” when they changed activities throughout the day. This involved teachers drawing children’s attention to something they may not have noticed (e.g., a specific body part such as their toes, an inconsequential object in the room such as a poster, a sound such as a ticking clock, or a scent such as paint). Children spent 1 min noticing and observing this before moving on to the next activity.	Focused attention and a state of calmness.
Mindful monkey, happy panda	This book was used to help children understand the concept of mindfulness. It was read to the children early in the term and served as a reference point for a range of activities such as drawing monkeys and pandas, and creating monkey and panda puppets for mindful play. Using the “monkey mind” as a metaphor for settling down.	Educational activity for understanding mindfulness.
Munch and crunch time – mindful eating	Mindful eating allows students to slow down and savor their food so that they pay more attention to what they are eating, rather than just eating quickly and not enjoying their food. This short snack break allows children to have a break and eat. It is standard across all prep schools. The first 2 min involved mindful eating. This exercise was led by the teacher and children were instructed to select a piece of fruit, to look at, smell, and taste it.	Focused attention, state of calmness, and cognitive flexibility
Control of the breath “blowing big bubbles”	Similar to belly breathing but children blow bubbles, teachers demonstrate that taking short sharp breathes not only make you feel more stressed and less relaxed but the bubbles aren’t as big and pop quicker. Children are then provided with a bubble mixture and are taught to take big, deep, slow breaths in order to blow big bubble while focusing there attention on their breath.	Focused attention, state of calmness, and cognitive flexibility
Watching clouds	During a cloudy day, the teacher gathers the children outside to lay on the grass outside their classroom and calmly watch the clouds. Students are encouraged to notice colors, shapes, and textures of the clouds. After a couple of minutes the teacher gathers the students in a circle again and discuss what the clouds looked like and how certain clouds may feel or made them feel. Once back inside students drew and/or painted the clouds they have seen as a classroom activity.	Focused attention, state of calmness, and cognitive flexibility
Rainbow walk	When walking outdoors, children were asked to look for something red, orange, yellow, green, blue, and purple. They were encouraged to keep going through the colors, in order, until the end of their walk. Once back in the classroom, the teacher invited students to talk about how the different colors made them feel and if some colors elicited stronger thoughts and emotions.	Focused attention, state of calmness, and cognitive flexibility
Glitter jar	Children created their own glitter jars which were then used to further teach controlled breathing and focused attention as they watched the glitter float to the bottom of their jars. This demonstrates that calm breathing helps busy minds to settle. Students were encouraged to use the glitter jar as an expression of how they are feeling at a point in time but also for others to understand how they are feeling.	Educational activity, focused attention, and state of calmness

#### Procedure

After obtaining approval by the Human Research Ethics Committee at the Griffith University, a regional Australian school was approached and the principal provided approval to conduct the study. Six classes were selected and initial consultation with the school principal and teachers occurred in Term 1, 2017 to discuss the overarching goals of the study. Teachers provided parents with an Information Sheet outlining the proposed study and a Consent Form to return to the classroom should they give written consent for their child to participate in the research study. Children, whose parents/guardians provided written consent, were then verbally provided with developmentally appropriate information regarding participation in the study and asked to circle a happy face if they consented to participate. All children received exposure to the intervention; only those children for whom there was Informed Consent were included in the data collection and analysis. The study was reviewed by the University and the State Education Department Human Ethical Review Committees and approval was obtained.

At the end of Term 2 the classroom teachers whose classes had been randomized to receive CalmSpace in Terms 3 and 4 received training in CalmSpace supported by the CalmSpace Program Manual. CalmSpace program teachers had a half-day training session and received weekly consultation with the lead researcher. During the training teachers were provided with the CalmSpace program manual which outlined the research base and theory behind the program, and included detailed scripts and materials for implementing the CalmSpace program. The half-day training included role-playing of the activities, discussion, and participation in a series of mindfulness exercises. Most teachers had little to no mindfulness experience. Teachers in the waitlist control condition received the half-day training in CalmSpace at the end of Term 3 and delivered the program in Term 4.

Child testing was conducted individually with a researcher in a quiet room separate from classroom activities in the first week of Term 3 (Pre), last week of Term 3 (Post1), and the last week of Term 4 (Post2). Children completed the Flanker Task followed by the DCCS on an IPad 2 with a 9.7 inch touch screen in accordance with the administration instructions provided by the National Institutes of Health Toolbox Administration Manual (National Institutes of Health and Northwestern University, 2018). Teachers completed the SDQ for each child via paper survey in their own time at the beginning and end of Term 3 and at the end of Term 4.

#### Intervention

##### Program summary

The mindfulness program, CalmSpace, was designed to enhance EFs by providing teachers with a core practice supplemented by a range of mindfulness-based activities that could be embedded within an existing school curriculum. This did not require departure from the core learning outcomes that are part of the Australian Curriculum. The CalmSpace activities were intended to support the development of EFs by (i) helping children to experience both a state of calmness and to develop skills that help return to such a state, thereby reducing the potential impact of bottom up processes such as anxiety and stress and (ii) help foster engagement in tasks that require focused attention in a calm state (Zelazo and Lyons, 2012). These combined elements are proposed to enhance the development of cognitive flexibility.

The construct of mindfulness was introduced to children in Week 1 by the reading of a children’s book, “Mindful Monkey, Happy Panda” (Alderfer and MacLean, 2011). This foundational activity served as the basis for a range of activities in which children were encouraged to be like Happy Panda and to become Mindful Monkeys. Children were given the opportunity to draw pictures of Mindful Monkey and make Monkey Puppets. They were further encouraged to be Mindful Monkeys and take mindful moments during transitions so the metaphor become an established term to describe being settled and calm. A core practice was undertaken three times a day in which children listened to a single resonant sound of a gong as they focused on the sound and slowed their breathe. This core practice was designed to induce a state of calmness and enabled the practice of focused attention. It was provided at key transition points across the school day: at the start of the day, on return from morning recess and lunch break.

In addition to the Core Practice, the program has 10 activities that are designed to promote EFs (Table 2). Additional activities addressed the two broad components associated with mindfulness training discussed above. A range of activities were provided that enabled children to experience a state of calmness: the early exercises focused on the “balloon belly” exercise, which is used widely in a number of mindfulness programs [e.g., MindUP (Hawn Foundation, 2008) and Smiling Mind (Smiling Mind, 2017)] and mindfulness introductions, where children were given the opportunity to learn to take slow deep breaths. This was then supplemented by a body scan exercise where children were able to practice mindful awareness of bodily sensations. Children were given the opportunity to play with bubbles and supported to experience deep breathing by the instruction to try to make the bubble as big as it could be with big gentle breaths. Children also made “glitter bottles” using plastic water bottles. These were used as visual aids to help with focusing and slowing their breath watching the glitter fall to the bottom of the bottle. The second component of mindfulness included in CalmSpace focused on developing awareness of everyday sensory experiences in order to train focused attention, and arguably enhance cognitive flexibility. For example, mindful eating during the 10 a.m. scheduled break becomes the “munch and crunch” time as children notice the fruit and the experience of eating, in other activities the children focused attention on their breath while imagining a bubble: requiring attention and executive control. Other activities that were longer exercises in developing and expanding sensory awareness was a cloud watching experience and a rainbow walk, each of which provide the opportunity to develop focused attention and foster cognitive flexibility (see Table 2 for a description of these activities).

The overall aim of the CalmSpace program design and activities was to provide multiple experiences of mindfulness that was woven into the existing school day. The purpose was to support the enhancement of attentional processes as this has been clearly linked to better academic performance and behavior [see Maynard et al. (2017) for a review of programs]. Importantly, the question as to whether mindfulness can also impact on behavior was further assessed using a child behavior screening questionnaire that included scales that measures attentional capacity and ability to follow directions and comply with adult requests, completed by teachers. Teachers were provided with a set of resources to implement the CalmSpace program, which included the children’s book Mindful Monkey, Happy Panda (Alderfer and MacLean, 2011), an A4 sized scrapbook for each child that was their own mindfulness diary, plastic bottles for a glitter bottle activity, bubble blowing bottles, and a gong. Approximate cost of resources per classroom was AUD100.

## Results

### Fidelity of Implementation

Teachers involved in the CalmSpace program implemented the program with high fidelity. Teachers in both the intervention and waitlist groups reported implementing the core practice 100% of the time (three times per day throughout the intervention period). Additionally, teachers in the intervention group reported embedding an average of two activities per day (range: 3–8 total activities) from Pre to Post1. From Post1 to Post2, teachers completed on average 2.5 (range: 3–9 total activities) activities throughout the day. Teachers in the waitlist group completed two (range: 3–7 total activities) activities from Post1 to Post2. Table 3 shows the average number of times an activity was delivered in each class per week.

**TABLE 3:** Average number of times a CalmSpace activity was delivered each week by class.

	**The gong**	**Munch and crunch**	**Balloon belly breathing**	**Body scan**	**Change of activity**	**Mindful monkey happy panda**	**Watching clouds**	**Control of breath “blowing big bubbles”**	**Rainbow walk**	**Glitter jar**
Prep A	15	4.7	3	1	0	0.5	1	1.5	1	1.2
Prep B	15	4.3	2.3	0.5	0.5	0.3	0.5	1.1	0.8	1
1/2 C	15	3.5	2	1	0.3	0.3	0.7	1	0.7	0.9
2A	15	3.8	2	1.3	0.4	0.4	0.3	1.1	0.5	0.7
1B	15	3.6	2.5	1.4	0.1	0.2	0.9	1.5	0.8	0.9

### Effects on Measures of Executive Functioning

A repeated measures analysis of covariance (ANCOVA; group: Intervention; waitlist control) was conducted to determine whether children showed an improvement in EFs following exposure to CalmSpace in their classroom compared to the waitlist control group (time: post-test 1; post-test 2). Covariates were baseline measures on the Flanker Task (reaction time; accuracy); DCCS; SDQ Total and SDQ subscales; and gender and Grade. Changes across the two time periods were examined using planned contrasts as (i) chi-squared tests revealed a gender difference between the CalmSpace group and waitlist group and (ii) potential differences due to grade may influence performance on tests of executive functioning. Baseline scores for the Flanker Task, DCCS Task, and SDQ total scores are summarized in Table 1. Results on the Group × Session interaction and planned contrasts for each measure and group are summarized in Table 4. In keeping with Cohen’s recommendations (1994) of reporting confidence intervals and the subsequent follow up paper by Sullivan and Feinn (2012) which highlighted the importance of including effect sizes and caution on over reliance of *p*-values, partial eta-square and Cohen’s *d* effects sizes are included in Table 4. Correlations between baseline behavioral measures of EF and the SDQ are provided in Table 5.

**TABLE 4:** Means and standard deviations (*SD*) of all the dependent variables (DVs) included in the study for each group at time Pre, Post 1, and Post 2.

**Measure**	**DV**	**Group**	**Baseline**	**Post 1**	**Post 2**	**Group × Time interaction *F*-value (${\text{η}}_{\text{p}}^{2}$)^1^**	**Planned contrasts (Cohen’s *d*)^2^**		
			**Mean (*SD*)**	**Mean (*SD*)**	**Mean (*SD*)**		**Baseline vs. Post 1**	**Post 1 vs. Post 2**	**Baseline vs. Post 2**
Flanker	Flanker score	Intervention	96.25 (11.26)	102.91 (10.20)	110.00 (10.04)	1.29 (0.02)	−6.99^∗∗^ (0.65)	−6.10^∗∗^ (0.77)	−9.07^∗∗^ (1.33)
		Waitlist	96.86 (12.42)	99.17 (13.60)	108.5 (10.41)		−1.26(0.18)	−5.66^∗∗^ (0.77)	−6.56^∗∗^ (1.02)
DCCS	DCCS score	Intervention	95.49 (13.28)	103.76 (10.41)	108.79 (10.08)	4.34^∗^ (0.05)	−6.84^∗∗^ (0.71)	−4.03^∗∗^ (0.48)	−7.60^∗∗^ (1.10)
		Waitlist	98.61 (16.29)	99.44 (11.93)	107.58 (10.39)		−0.41(0.06)	−5.69^∗∗^ (0.73)	−3.82^∗∗^ (0.66)
SDQ	Total	Intervention	7.76 (6.59)	4.48 (5.20)	2.93 (4.31)	11.80^∗∗^ (0.12)	5.22^∗∗^ (0.53)	4.12^∗∗^ (0.32)	6.37^∗∗^ (0.85)
		Waitlist	5.86 (5.34)	6.26 (5.10)	3.86 (4.27)		−0.68(0.08)	4.86^∗∗^ (0.51)	2.89^∗^ (0.41)
	Emotional symptoms	Intervention	1.09 (1.85)	0.67 (1.727)	0.44 (1.56)	1.98 (0.05)	2.38^∗^ (0.22)	2.12^∗^ (0.14)	3.33^∗^ (0.37)
		Waitlist	0.57 (1.01)	0.83 (1.18)	0.37 (0.77)		−1.39(0.24)	2.36^∗^ (0.46)	1.07(0.22)
	Conduct problems	Intervention	1.09 (1.80)	0.72 (1.25)	0.39 (0.90)	3.13 (0.04)	1.68(0.22)	3.37^∗^ (0.30)	2.96^∗^ (0.48)
		Waitlist	0.77 (1.37)	1.00 (1.61)	0.57 (0.917)		−1.68(0.15)	2.27^∗^ (0.33)	1.27(0.17)
	Hyperactivity/attention	Intervention	4.00 (3.29)	1.81 (2.32)	1.43 (2.19)	10.41^∗∗^ (0.11)	6.84^∗∗^ (0.74)	2.23^∗^ (0.17)	7.10^∗∗^ (0.90)
		Waitlist	3.29 (2.54)	3.69 (2.54)	2.37 (2.44)		−1.28(0.16)	5.60^∗∗^ (0.53)	2.89^∗^ (0.37)
	Peer problems	Intervention	1.57 (1.93)	1.28 (1.49)	0.67 (1.10)	0.41 (0.01)	1.27(0.16)	3.83^∗∗^ (0.47)	3.83^∗∗^ (0.57)
		Waitlist	1.23 (1.59)	0.74 (1.20)	0.54 (0.98)		2.27^∗^ (0.35)	1.19(0.18)	2.76^∗^ (0.52)
	Prosocial	Intervention	6.78 (3.01)	7.78 (2.90)	8.39 (2.74)	4.22^∗^ (0.05)	−3.26^∗^ (0.32)	−3.11^∗^ (0.22)	−4.77^∗∗^ (0.55)
		Waitlist	7.94 (2.51)	8.20 (2.26)	9.00 (1.59)		−0.88(0.11)	−4.28^∗∗^ (0.41)	−3.58^∗^ (0.50)

*The columns on the right show results of Group × Time interactions and planned contrasts performed between Pre vs. Post 1, Pre vs. Post 2, and Post 1 vs. Post 2 scores for each group. ^∗^*p* < 0.05; ^∗∗^*p* < 0.001; DCCS, Dimensional Change Card Sort; SDQ, Strengths and Difficulties Questionnaire. ^1^Effect size ($\eta_{p}^{2}$) interpreted as: small – 0.01; medium – 0.06; and large – 0.14. ^2^Effect size (Cohen’s *d*) interpreted as: small – 0.2; medium – 0.5; and large – 0.8.*

**TABLE 5:** Baseline correlations between Executive Function Tasks and Strengths and Difficulties Questionnaire.

			**Flanker**			**DCCS**	**SDQ**					
			**Flanker score**	**Flanker reaction time**	**Flanker accuracy**	**DCCS score**	**Emotion symptoms**	**Conduct problems**	**Hyperactivity/inattention**	**Peer problems**	**Prosocial**	**Total**
Flanker	Flanker score	*r*		−0.500^∗∗^	0.424^∗∗^	0.421^∗∗^	−0.065	−0.054	−0.116	−0.117	0.121	−0.122
	Flanker reaction time	*r*	−0.500^∗∗^		−0.525^∗∗^	−0.251^∗^	−0.003	0.003	0.016	0.248^∗^	−0.075	0.080
	Flanker accuracy	*r*	0.424^∗∗^	−0.525^∗∗^		0.288^∗∗^	0.150	−0.059	−0.078	−0.208	0.194	−0.076
DCCS	DCCS score	*r*	0.421^∗∗^	−0.251^∗^	0.288^∗∗^		−0.137	−0.117	−0.229^∗^	−0.188	0.322^∗∗^	−0.233^∗^
SDQ	Emotion symptoms	*r*	−0.065	−0.003	0.150	−0.137		0.090	0.115	0.300^∗∗^	−0.197	0.425^∗∗^
	Conduct problems	*r*	−0.054	0.003	−0.059	−0.117	0.090		0.697^∗∗^	0.682^∗∗^	−0.697^∗∗^	0.831^∗∗^
	Hyperactivity/inattention	*r*	−0.116	0.016	−0.078	−0.229^∗^	0.115	0.697^∗∗^		0.568^∗∗^	−0.706^∗∗^	0.871^∗∗^
	Peer problems	*r*	−0.117	0.248^∗^	−0.208	−0.188	0.300^∗∗^	0.682^∗∗^	0.568^∗∗^		−0.705^∗∗^	0.830^∗∗^
	Prosocial	*r*	0.121	−0.075	0.194	0.322^∗∗^	−0.197	−0.697^∗∗^	−0.706^∗∗^	−0.705^∗∗^		−0.788^∗∗^
	Total	*r*	−0.122	0.080	−0.076	−0.233^∗^	0.425^∗∗^	0.831^∗∗^	0.871^∗∗^	0.830^∗∗^	−0.788^∗∗^	

*^∗^*p* < 0.05; ^∗∗^*p* < 0.001; DCCS, Dimensional Change Card Sort; SDQ, Strengths and Difficulties Questionnaire; *r*, Pearson correlation.*

#### Flanker Task

Repeated measures ANCOVA revealed a significant main effect of Time for Flanker Score, *F*(1,83) = 15.85; *p* < 0.001, and a significant main effect of Group, *F*(1,83) = 4.929; *p* = 0.029. There was no significant Time by Group interaction for Flanker Score, *F*(1,83) = 1.29; *p* = 0.260.

#### DCCS Task

**FIGURE 2:**
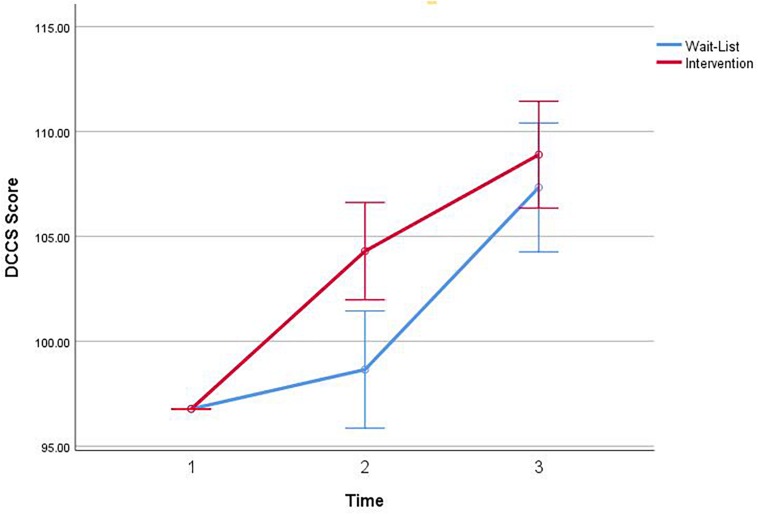
Interaction of group × time on the dimensional card change sort (DCCS) task total score.

There was a significant main effect of Time, *F*(1,83) = 15.44, *p* < 0.001, and a significant Group by Time interaction for DCCS Score, *F*(1,83) = 4.34, *p* = 0.040 (Figure 2). There was a significant Group effect, *F*(1,83) = 4.37, *p* = 0.040. Simple effects of the significant interaction of DCCS Score revealed that the intervention group (*M* = 103.76; *SD* = 10.41) scored significantly higher at Post1 than the waitlist group (*M* = 99.44; *SD* = 11.93) at Post1, *F*(1,83) = 8.94, *p* = 0.004.

Planned contrasts for Pre and Post1 revealed significant improvements for the intervention group in DCCS score, *t*(83) = −6.84, *p* < 0.001. There were no significant contrasts found for the waitlist group. Post1 and Post2 planned contrasts revealed a significant improvement in DCCS score for both the intervention, *t*(83) = −4.03, *p* < 0.001, and waitlist, *t*(83) = −5.69, *p* < 0.001. Planned contrasts for Pre and Post2 found significant improvements in DCCS score for the intervention group, *t*(83) = −7.60, *p* < 0.001, and waitlist group *t*(83) = −3.82, *p* < 0.001.

### Teacher Report of Child Behavior

#### SDQ Total Score

**FIGURE 3:**
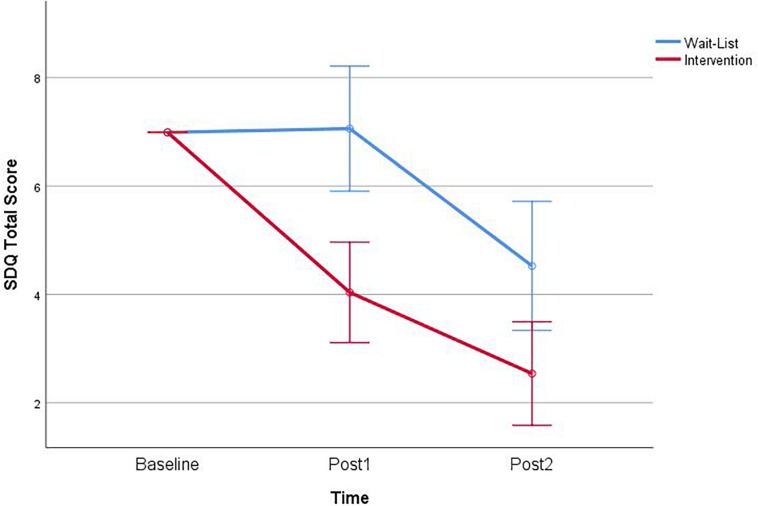
Interaction of group × time on the strengths and difficulties (SDQ) teacher report total score.

There was no significant effect of Time, *F*(1,83) = 0.36, *p* = 0.548, and no significant effect of Group, *F*(1,83) = 0.71, *p* = 0.403. However, there was a significant interaction between Group and Time, *F*(1,83) = 11.80, *p* = 0.001 (Figure 3). Simple effects of the interaction revealed that the intervention group was significantly higher in SDQ total score than the waitlist group at Post1, *F*(1,83) = 4.12, *p* = 0.045.

Follow-up planned contrasts for Pre versus Post1 revealed significantly improved teacher ratings for the intervention group, *t*(83) = 5.22, *p* < 0.001. There were no significant contrasts for the waitlist group. Planned contrasts of Post1 and Post2 revealed significantly improved teacher ratings for the intervention group, *t*(83) = 4.12, *p* < 0.001, and improved ratings for the waitlist group *t*(83) = 4.86, *p* < 0.001. Pre and Post2 planned contrasts showed significant improvements in the intervention, *t*(83) = 6.37, *p* < 0.001, and waitlist group, *t*(83) = 2.89, *p* = 0.007.

#### Emotional Problems Subscale

Analysis of the SDQ emotion subscale revealed a non-significant main effect of Time, *F*(2,83) = 2.85, *p* = 0.064, and a non-significant effect of Group, *F*(1,83) = 0.10, *p* = 0.759. There was also no significant interaction effect, *p* = 0.145, was found.

#### Conduct Problems Subscale

Analysis of the conduct subscale of the SDQ found a non-significant main effect of Time, *F*(1,83) = 2.22, *p* = 0.140. No significant main effect for Group, *F*(1,83) = 0.32, *p* = 0.576, and interaction effect, *F*(1,83) = 3.13, *p* = 0.081, was found.

#### Hyperactivity and Inattention Subscale

**FIGURE 4:**
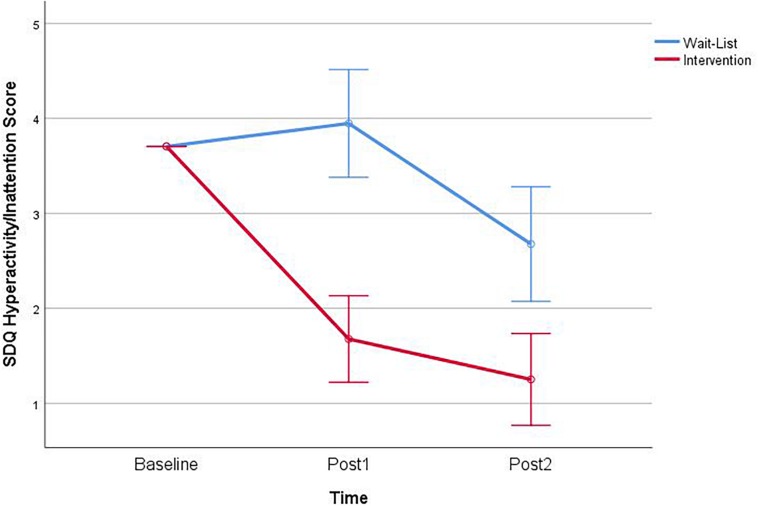
Interaction of group × time on the strengths and difficulties (SDQ) teacher report hyperactivity and inattention subscale.

Analysis of the hyperactivity and inattention subscale revealed a non-significant main effect of Time, *F*(1,83) = 0.36, *p* = 0.548, and a significant interaction between Group and Time, *F*(1,83) = 10.41, *p* = 0.002 (Figure 4). There was significant effect of Group, *F*(1,83) = 7.56, *p* = 0.07. Follow-up simple effects of the interaction finding for the hyperactivity and inattention subscale score revealed that the CalmSpace children (*M* = 1.81; *SD* = 10.20) were rated better than the waitlist group (*M* = 3.69; *SD* = 3.69) at Post1, *F*(1,83) = 14.59, *p* < 0.001, as having greater attention and concentration skills.

Follow-up planned contrasts for Pre vs. Post1 revealed significantly improved teacher ratings for the intervention group, *t*(83) = 6.84, *p* < 0.001. There were no significant contrasts for the waitlist group. Planned contrasts of Post1 and Post2 revealed significantly improved teacher rating for the intervention group, *t*(83) = 2.23, *p* = 0.030, and the waitlist group only *t*(83) = 5.60, *p* < 0.001. Pre and Post2 planned contrasts showed significant improvements in the intervention, *t*(83) = 7.10, *p* < 0.001, and the waitlist group, *t*(83) = 2.89, *p* = 0.007.

#### Peer Relationship Problems Subscale

Repeated measures ANOVA revealed a non-significant main effect of time, *F*(1,83) = 0.22, *p* < 0.640. There was a significant main Group effect, *F*(1,83) = 5.51, *p* = 0.021, but no interaction of Group and Time, *F*(1,83) = 0.41, *p* = 0.525.

#### Prosocial Behavior Subscale

**FIGURE 5:**
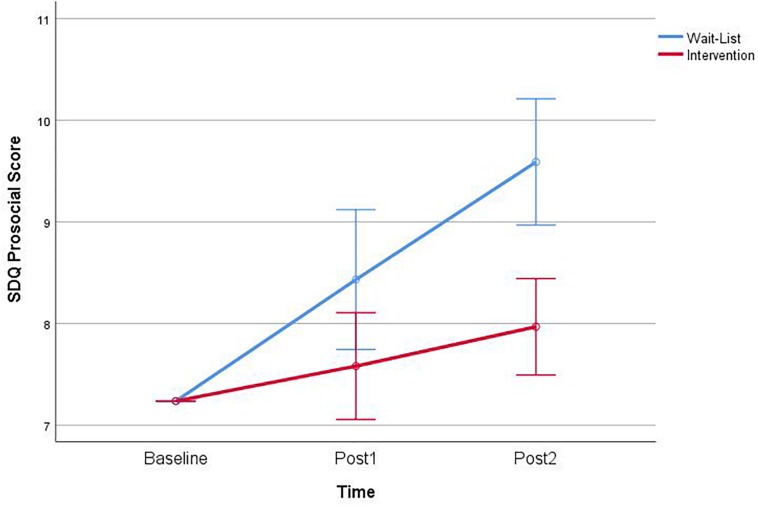
Interaction of group × time on the strengths and difficulties (SDQ) teacher report prosocial.

A significant effect of Time, *F*(1,83) = 22.16, *p* < 0.001, and Group *F*(1,83) = 8.25, *p* = 0.005, was found. There was a significant Group by Time interaction effect, *F*(1,83) = 4.22, *p* = 0.043, was found (Figure 5). Follow-up simple effects interaction found that children in the intervention group (*M* = 9.00, *SD* = 1.59) were rated higher in prosocial behavior than the waitlist group (*M* = 8.36, *SD* = 2.75) at Post2, *F*(1,83) = 13.26, *p* < 0.001.

Follow-up planned contrasts for Pre vs. Post1 revealed significant improved teacher ratings for the intervention group, *t*(83) = −3.26, *p* = 0.002. There were no significant contrasts for the waitlist group. Planned contrasts of Post1 and Post2 revealed significantly improved teacher rating for the intervention group, *t*(83) = −3.11, *p* = 0.003, and the waitlist group only *t*(83) = −4.28, *p* < 0.001. Pre and Post2 planned contrasts showed significant improvements in the intervention, *t*(83) = −4.77, *p* < 0.001, and the waitlist group, *t*(83) = −3.58, *p* = 0.001.

## Discussion

While there has been growing evidence for the effectiveness of school-based mindfulness programs to enhance EFs, there has been relatively little evaluation of the potential to improve EFs in children in the early school years (Preparatory/Kindergarten, Grades 1 and 2). The results from this study provide evidence that a series of activities drawing from key mindfulness constructs can be integrated within an existing primary school curriculum. Further, that relative to children in a waitlist control condition, those children who participated in the mindfulness program, CalmSpace, showed improvements in measures of inhibitory control and cognitive flexibility. There were also significant gains in measures of behavior, most notably in attentional control processes. The mindfulness program, CalmSpace, was specifically designed to be embedded within an existing school curriculum and delivered by teachers. Teachers reported being able to embed a core practice and mindfulness-based activities consistently across the school day with fidelity.

### Effects on Executive Functioning in Children in the Early School Years

Previous research has shown that EFs can be improved through regular mindfulness training in both adults and older children (Davidson et al., 2003; Jha et al., 2007; Schonert-Reichl and Lawlor, 2010; Hölzel et al., 2011; Schonert-Reichl et al., 2015). However, persuasive evidence suggests that early childhood is a critical time for the development of EFs, a key requirement for social and academic success in early childhood (Anderson et al., 2011). Therefore, interventions aimed at improving developing EFs in young children are warranted. CalmSpace provided an opportunity for young children to enhance their capacity to attend and focus their attention, and to develop the key skills involved in behavioral inhibition by embedding mindfulness practice throughout the school day. Further, children were provided with an opportunity to acquire an understanding of the concepts of mindfulness through a range of age-appropriate and fun activities that had educational value, e.g., reading Happy Monkey, Mindful Panda, taking nature walks, and drawing and writing in scrapbooks to describe experiences of being calm.

The results from two robust measures of EF in young children were encouraging. After controlling for baseline scores, students showed significant improvement on the DCCS, which measured changes in cognitive flexibility. Like the Flanker task, the DCCS scoring is based on a combination of accuracy and reaction time. Results demonstrate that students in the CalmSpace program were able to outperform students in the waitlist group by being able to shift attention effectively and respond to new stimuli while keeping certain rules in mind. This finding is supported by the CalmSpace students scoring significantly higher than the waitlist group at the end of Term 3 and with the CalmSpace students demonstrating improved ability to flexibly respond at each time-point. Supporting these effects of the program, children in the waitlist group significantly improved following participation in CalmSpace in Term 4. Notably, the significant difference between those on the waitlist and those receiving CalmSpace in Term 3 suggests that this improvement may have been enhanced by embedding the mindfulness exercises in the school curriculum (Zelazo, 2006).

### Effects on Teacher-Reports of Behavior

The teacher version of the SDQ was used to capture teachers’ perceptions of five subscales: (1) emotional symptoms, (2) conduct problems, (3) hyperactivity/inattention, (4) peer problems, and (5) prosocial behavior, summing to a total difficulties score. Teachers’ reports of children’s behavior indicated that a number of children were scoring in the clinical range at baseline (Table 1). Marked improvements in teacher reports of children’s behavior mirrored the findings from the direct measures of EFs. There was a large improvement in Total Scores on the SDQ across time demonstrating significant improvement in those children participating in CalmSpace at the end of Term 3 compared to children in the waitlist control condition.

Further analysis of the subscale results indicated that this effect on Total Scores was primarily the result of improvements in the Hyperactivity/attention subscale, with a moderate to large effect (Cohen, 1988). This is particularly noteworthy as the focus of CalmSpace is to help children improve EFs and learn to direct their attention through the practice of intentional self-regulation of attention during routine activities (e.g., mindful eating, watching clouds). Previous research has found that children who exhibit hyperactivity benefit from EF intervention (Reid et al., 2005). By teaching children mindfulness, it is suggested that impulse-control can be improved and emotional reactivity decreased (Thompson and Gauntlett-Gilbert, 2008; Viglas and Perlman, 2017). Children who lack inhibitory control are typically characterized as impulsive (Schachar and Tannock, 1993). Therefore, our findings are consistent with research showing the association between mindfulness and inhibitory control in children (Oberle et al., 2011). Improvements in attention and concentration have also been found in previous studies of children with attention problems (e.g., Semple et al., 2010) and of children’s capacity to regulate attention (Rani and Rao, 1996).

### Program Implementation and Fidelity

A growing focus on the delivery of mindfulness programs by teachers underscores the need to ensure that such programs can be included into normal, day-to-day teaching. This requires a clear description of the program’s core components at the outset and subsequent measurement of adherence and dose. Two further components that should be measured are quality of the program delivery and responsiveness of the program recipients (Gould et al., 2015). The present study measured both adherence to core components and dosage. Teachers reported that the gong was used at the end of every major transition for core practice, and that there was strong utilization of the additional activities with on average two to two and a half used each day.

Teachers were also receptive to the weekly support sessions provided, and made several anecdotal comments about their use of the program during these sessions. Some of the comments from teachers included: “There is a noticeable calmness within the classroom, so much more learning happens and children are quicker to start a task and engage with it longer”; “The gong is very effective at starting the day off in a calm manner and really helps students develop a routine to begin work quicker”; “Mindful eating really helps with the students eating habits, they don’t just devour their food now but take time to eat slowly. Some even get full before they finish all their food now”; “We had a health nurse come into the class and she commented on how amazing the children were and how calm they were when she was with them as a group. They were very attentive and engaged.” These comments, although anecdotal, show that teachers were able to see notable differences among their students and despite their heavy workload could adopt this program into routine classroom activities.

Interestingly, the comments also touch on the transferability of the skills learnt in class to outside the classroom. The comment from the health nurse regarding the attentiveness of the students suggests that not only did teachers observe a difference but so did an external person who had no knowledge that the CalmSpace program was being conducted at the school. Further, parents reportedly made comments to teachers about the calmness of their children, and that they were more adept at self-regulation. Parents reportedly requested further information about the program so they could support their children at home. It would thus be beneficial for future research to evaluate parents’ perceptions of their children’s behavior to determine if the program had similarly positive effects outside of the school environment.

### Strengths and Limitations

The major strength of this study is the ecological validity of the delivery of the intervention. Teachers were able to integrate a series of developmentally appropriate mindfulness activities into an existing classroom routine. It is reasonable to consider whether a trained instructor would provide a more rigorous and theoretically informed delivery of a mindfulness program. However, reliance on external experts reduces the potential for dissemination and sustainability. A substantial number of mindfulness-based educational programs are delivered by teachers (e.g., approximately 30% in the recent meta-analysis reported by Maynard et al., 2017). This meta-analysis found similarity in outcomes regardless of, *inter alia*, teacher vs. expert in delivery. The actual content of the CalmSpace program enhances potential feasibility. The program was compiled by a careful reading of the literature and review of related programs and drew together a range of resources that are in the public domain. Thus, wider dissemination and adaption can be easily considered, and teachers can be encouraged to try to assemble a similar set of resources. The core components of a mindfulness program could also be reconsidered: there are a number of programs that explicitly address empathy or compassion within the context of a mindfulness program. Adding this as the third mindfulness component is worthy of consideration. Measuring executive functioning in this younger age group is challenging. The two subtests selected from the NIH Toolkit are well-validated measures of two key constructs of EF: behavioral inhibition and cognitive flexibility. It may be worth considering adding a further measure that is specific to working memory such as the List Sorting Working Memory subtest.

It is important to note that two of the four implementation components described by Gould et al. (2015) were not included in the implementation of CalmSpace for the present study. The present implementation of the CalmSpace program satisfied the first two components: teachers adhered to the core components of the program, and implemented it as designed. With respect to dosage, teachers completed a daily checklist of the activities that were delivered throughout the day. However, the quality or program delivery and responsiveness were not measured. While the researchers had weekly consultations with the teachers delivering the program and spent time in the classroom to ensure that the activities were being implemented appropriately, these two components were not formally assessed. The representativeness of the sample also needs to be considered. Figure 1 shows that 80% of parents/carers provided consent. It is not possible to say definitively that there were no differences between those who consented and those who did not (as with any controlled study there is no data on participant characteristics for those who do not consent). It is always possible that there were some systematic differences but the high uptake rate for the current study does provide some confidence that this was a representative sample of children attending the school. A further limitation is the potential for bias in collection of the data. The researcher who collected the data (PJ) was also directly involved with supporting the teachers to implement CalmSpace. Thus, consideration of the potential for inadvertent bias toward finding an intervention effect needs to be considered. In the current study, EFs were assessed using a structured assessment process administered via IPad and subsequently scored using an inbuilt scoring algorithm. Although the delivery of the EF measures is through the NIH toolbox in a structured assessment process, it is possible that the researcher may implicitly affect performance of the participants before and during testing. Therefore, it is impossible for the complete elimination of potential researcher bias while participants undertook the NIH toolbox and hence a limitation of this study. As teachers were the primary deliverers of CalmSpace it was also not possible for them to be unaware of the group to which children were allocated. They were keen to be involved and expressed ongoing commitment to the process of engaging in mindfulness within their classrooms. These are highly encouraging sentiments but raise concerns regarding potential underreporting of children’s behavior problems. It is also possible that children’s behavior did not improve but rather, teachers’ tolerance of poor behavior increased and reactivity to it decreased.

There is also a growing literature on the effectiveness of mindfulness-based interventions on teacher well-being (Emerson et al., 2017) which suggests that effects for educators across a range of settings are generally positive. As such, it is possible that teachers developed greater capacity to tolerate and manage hyperactivity and inattention. The potential impact of engaging in and delivering mindfulness programs on teacher well-being should be included in future research.

A final limitation relates to the study design. As with any waitlist-controlled trial only short-term benefits can be ascertained. Longer term studies are required to ensure that the gains found in this study were not simply a slight escalation in a normal developmental process. Further, as no parent measure was used it is not possible to ascertain if the students practiced any of the activities that were taught in class at home. Parents who observed improvements in their children’s behavior and self-regulation may have been more likely to enquire about the activities and possibly further support their children outside of the classroom, thereby reinforcing positive effects. This is an area for potential future research as previous studies have combined classroom-based mindfulness programs with parent inclusion (e.g., Semple et al., 2010).

## Conclusion

CalmSpace was designed to improve EFs in young children from Preparatory Year through to Grade 2. This small study is the first to examine the effectiveness of such a program using a range of classroom activities that complement the day-to-day routine and curriculum. The findings indicate that implementing the CalmSpace program can lead to improvements in EFs and attention in young children. Despite some limitations, this study provides promising evidence that the inclusion of brief and targeted mindfulness activities throughout the school day may represent a value-added component to the regular school curriculum that can result in benefits for the students.

A carefully designed randomized control trial is the next logical step in determining the effectiveness of the CalmSpace program. This research should include a diverse group of students from multiple schools and areas to ascertain if effectiveness of the program can be found across a range of students. As students spend most of their time at home consideration should be made to the inclusion of a parent measure or to the impact of home life on the program. In addition, evaluation of the quantity of activities that are delivered by the classroom teachers (i.e., the “dose” of the intervention) will be important to consider whether children benefit more with greater participation in CalmSpace. Further, gathering a measure of personal change in the teachers to inform additional factors that may contribute to the change in students is warranted. Regardless, future research should continue to contribute to the evidence base that will help all children to thrive and reach their potential.

## Data Availability

The raw data supporting the conclusions of this manuscript will be made available by the authors, without undue reservation, to any qualified researcher.

## Ethics Statement

This study was carried out in accordance with the recommendations of the Australian National Statement on Ethical Conduct in Human Research and the Griffith University Human Research Ethics Committee. The protocol was approved by the Griffith University Human Research Ethics Committee. All subjects gave parent written informed consent and assent in accordance with the Declaration of Helsinki.

## Author Contributions

PJ, SD, and MW contributed ideas to the mindfulness program, CalmSpace, and writing of the final manuscript. PJ provided the training and implementation support to the teachers. PJ and SD designed the waitlist controlled trial and selected measures and contributed to the data analysis.

## Conflict of Interest Statement

The authors declare that the research was conducted in the absence of any commercial or financial relationships that could be construed as a potential conflict of interest.
